# Occurrence and Phylogenetic Analysis of DWV in Stingless Bee (Apidae sp.) in China: A Case Report

**DOI:** 10.3389/finsc.2021.748074

**Published:** 2021-11-12

**Authors:** Lina Zhang, Yanchun Deng, Hongxia Zhao, Ming Zhang, Chunsheng Hou

**Affiliations:** ^1^Institute of Apicultural Research, Chinese Academy of Agricultural Sciences (CAAS), Beijing, China; ^2^Key Laboratory of Pollinating Insect Biology, Ministry of Agriculture and Rural Affairs, Beijing, China; ^3^Graduate School of Chinese Academy of Agricultural Sciences, Beijing, China; ^4^Guangdong Key Laboratory of Animal Conservation and Resource Utilization, Guangdong Public Laboratory of Wild Animal Conservation and Utilization, Institute of Zoology, Guangdong Academy of Sciences, Guangzhou, China

**Keywords:** honey bee viruses, deformed wing virus, stingless bee, Apidae sp, pathogen spillover

## Abstract

Honey bees play a vital role in providing pollination services for agricultural crops and wild flowering plants. However, the spillover risk of their pathogens to other pollinators or wild insects is becoming a cause for concern. There is some evidence that stingless bees can carry honey bee viruses, but little is known about the presence of honey bee viruses in stingless bees in China. Here, we investigate the occurrence of major honey bee pathogens including bacteria, fungi, and viruses in stingless bees (Apidae: sp.). Our results show that the stingless bees (Apidae: sp.) were mainly infected with DWV-A, but no DWV-B and DWV-C. Phylogenetic analysis on fragments of *lp, RdRp*, and *VP3* of DWV-A indicated that genetic variation in VP3 might an important indicator for host-specific viruses, but it requires further study. Our results indicated that DWV-A is not only the major strain of virus currently circulating in managed bee colonies in China and globally, but in stingless bee species as a whole.

## Introduction

Honey bees play an important role in flowering plants in the agri-ecosystem, but viral diseases pose a severe threat to honey bee population growth (1). It was reported that more than 20 bee viruses were identified that affected the health and survival of bees (2). Among them, eight common viruses were *Israel acute paralysis virus* (IAPV), *Deformed wing virus* (DWV), *Black queen cell virus* (BQCV), *Sacbrood virus* (SBV), *Chronic bee paralysis virus* (CBPV), *Acute bee paralysis virus* (ABPV), *Kashmir bee virus* (KBV), and *Kakugo virus* (KV) (3–10).

DWV is considered the most prevalent bee virus worldwide due to its close relation to colony decline induced by interactions between *Varroa destructor* and DWV (11). DWV is a positive single-stranded RNA virus and its genome is about 10 kb in length (12). Currently, three master variants of DWV have been identified: type A (4), type B (13), and type C (14). Initially, only one strain, DWV, was identified in honey bee colonies around the world. Then, DWV-B, previously designated as *V. destructor virus*-1 (VDV-1), came to be considered as one viral cloud with DWV-A (14). DWV-C is a recently described strain and its impact is still unknown (14). DWV-A is currently the most prevalent genotype and is closely related to colony decline (15–17). The titer of DWV-B is inhibited significantly when DWV-A is a presence (18, 19). In addition, the co-existence of the three genotypes of DWV is a frequent occurrence, leading to their recombination (14, 20–22). Although KV was identified in aggressive workers in 2004 (23), subsequent studies did not find that KV was a primary virus of *Apis mellifera* colonies (24, 25).

DWV is causing growing concern because it can infect and replicate in multiple invertebrates including bumblebees, solitary bees, wasps, and ants (26, 27). This suggests that DWV is spreading to other insect species, especially wild bee species, and poses a potential threat to their population growth (28–32). It was reported that virulent pathogens and parasites can spill over from cultivated bees to wild bees such as the stingless bee (29, 31, 33), but whether the bee viruses were a presence in stingless bee in China was still unknown.

In this study, we attempted to test whether common honey bee pathogens were present in the stingless bee, Apidae sp. We found that two strains of DWV (DWV-A and DWV-A/KV) were found in stingless bees collected in the Yunnan province of China. Phylogenetic analysis showed that DWV-A or DWV-A/KV from the stingless bees was close to that found in *A. mellifera*. This suggested that DWV in these stingless bees was most likely obtained from *A. mellifera*, but it needs to be further confirmed.

## Materials and Methods

### Samples Collection

The samples of stingless bees *Apidae* sp. were collected in the field in Yunnan province in December 2019. Live bee samples alive were put into a small cage and transported to the laboratory at −80°C until use. We identified the stingless bee species using PCR amplification with the universal primers (LCO-1490 and HCO-2198) previously used (34).

### DNA and RNA Extraction

The total DNA was extracted from pooled *Apidae* sp. workers (~5-10 bees) with the TissuePrep homogenizer (Gening Scientific, Beijing, China). Briefly, bee samples were added 1 ml of lysis buffer and 10 μl of proteinase K and then heated at 65°C in a water bath for 30 min. Subsequently, the supernatant was taken after low-temperature high-speed centrifugation and added to an equal volume of phenol: chloroform: isoamyl alcohol (25:24:1), and then centrifuged at 4°C. Finally, the precipitation was washed with 75% ethanol, and then 30-50 μl ddH_2_O was added to dissolve the DNA.

The total RNA was isolated from pooled stingless bee samples using TRIzol Kit (Invitrogen, USA) according to the manufacturer's protocol with the TissuePrep homogenizer (Gening Scientific, Beijing, China). The obtained RNA was dissolved in 20 μl of sterile water and stored at −80°C prior to use. The quantity and purity of the RNA were measured using a Nanodrop spectrophotometer (Thermo Scientific, Beijing, China). The cDNA samples were used as templates for PCR amplification with specific primers corresponding to target genes.

### PCR Detection

The primer sequences and the target size of amplification for honey bee common pathogens were provided in Supplementary Tables 1, 2. PCR amplification of non-virus pathogens of honey bee was performed in a 20 μl volume containing template DNA, 2×GoTaq Mix (Promega, USA), and corresponding primers as shown in Supplementary Table 1. PCR amplification was performed for 2 min at 94°C, 30 s at 94°C, 30 s at 55°C, 30 s at 72°C for 32 cycles, and 5 min at 72°C.

For the detection of honey bee RNA viruses, the extracted RNA was used to synthesize cDNA using GoScirpt reverse transcriptase (Promega, Madison, WI, USA). The initial cycle was performed for reverse transcription at 80°C for 30 min, and then followed by 1 min at 95°C; 33 cycles of 30 s at 94°C, 30 s at 55°C, and 72°C for 1 min; a final extension of 10 min at 72°C; and cooling to 4°C. The PCR amplification products were separated in a 2% agarose gel stained with GV II (Biomec, China) and photographed with a fluorescent biological-image analysis system (Furi, China). The product size was determined with a 100-bp ladder.

Then, we focused on the amplification on four fragments, part of DWV, RdRp, Lp, and VP3 using the corresponding primers (Supplementary Table 3) with an initial denaturation for 3 min at 98°C, 10 s at 98°C, 20 s at 55°C, 30 s at 72°C for 30 cycles, and 10 min at 72°C.

### Phylogenetic Analysis

The target products were purified and sent to be sequenced by Sangon corporation (Sangon, Shanghai, China). The sequencing results were compared with those sequences from GenBank using NCBI (National Centre for Biotechnology Information) BLAST (Basic Local Alignment Search Tool) to determine the similarity. Phylogenetic trees were constructed based on the nucleotide sequences using the maximum likelihood method (Maximum Likelihood) to infer its evolution process as run on MEGA 7 software (35, 36). The 40 nucleotide sequences were selected and their specific information is shown in Supplementary Table 4. The sequences of RdRp, Lp, and VP3 of DWV-A from the Apidae sp. were obtained under the accession numbers of MT899243 (DWV-A), MT899244 (Lp), MT899245 (RdRp), MT899247 (VP3), as well as MT899246 (DWV-A/KV).

## Results

### Virus Occurrence

We conducted a PCR analysis with stingless bee Apidae sp. collected in wild and screened for the presence of 10 viruses and 6 bacterial or fungal pathogens of honey bees (Supplementary Table S1). Only two strains of DWV, DWV-A and DWV-A/KV, were found and were used to further study the phylogenetic relation among the known strains from bees, *Varroa destructor*, and wasp species around the world.

### Phylogenetic Analysis

**Figure 1:**
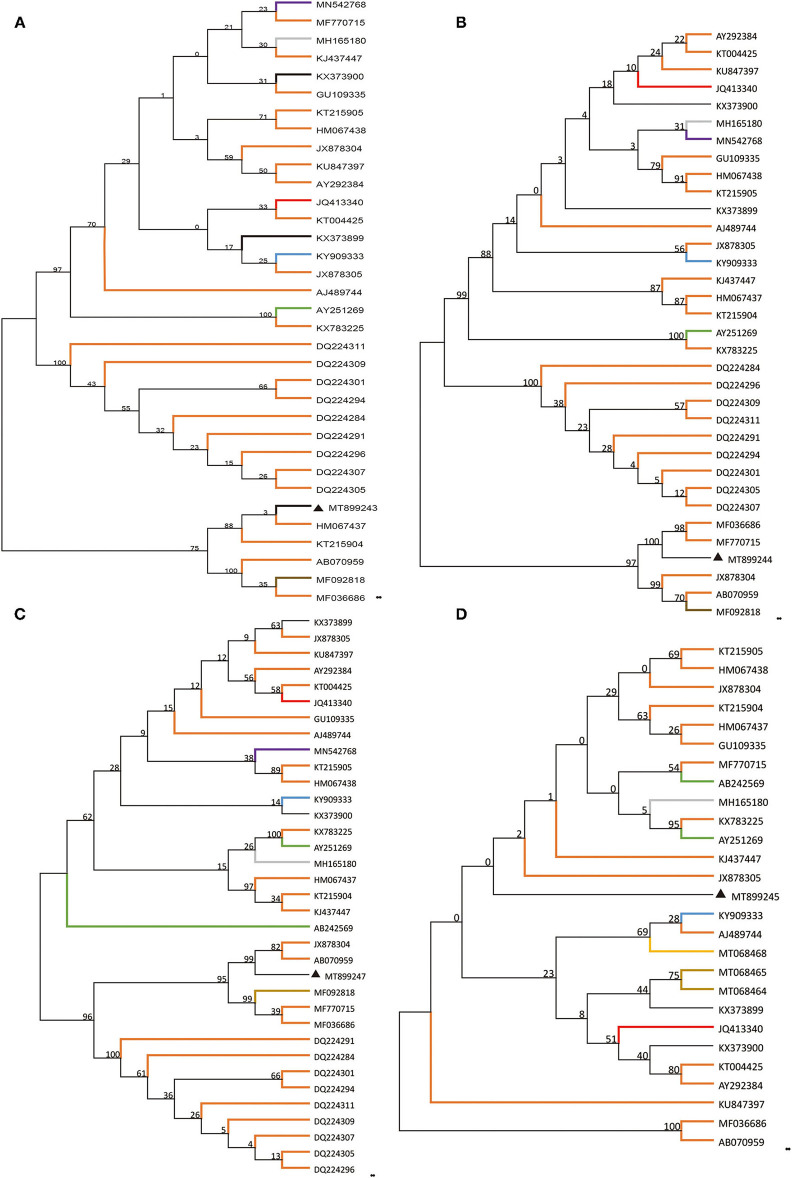
Phylogenetic reconstruction of four fragments of DWV showing host and geographic structure. The host included different bee, wasp, and ant species as well as *Varroa destructor*. Phylogenetic analysis on four genomic fragments, part of DWV-A **(A)**, Lp **(B)**, VP3 **(C)**, and RdRp **(D)**. The maximum likelihood method (ML) and bootstrap resampling (1,000 repetitions) were used to construct a phylogenetic tree. The number on each branch of the phylogenetic tree represents the bootstrap value (1,000 repetitions), and the branches with different colors indicate different species infected by different DWV strains. The different colors indicate DWV strains from a different host. *Apis mellifera, Apis cerana, Vespa crabro, Varroa destructor*, vespids wasp, ant, *polistes chinesis, Vespula vulgaris*, stingless bees, bombus, and unknown host are indicated in orange, gray, blue, green, brown, purple, yellow, khaki, red, gray-blue, and black, respectively.

**Figure 2:**
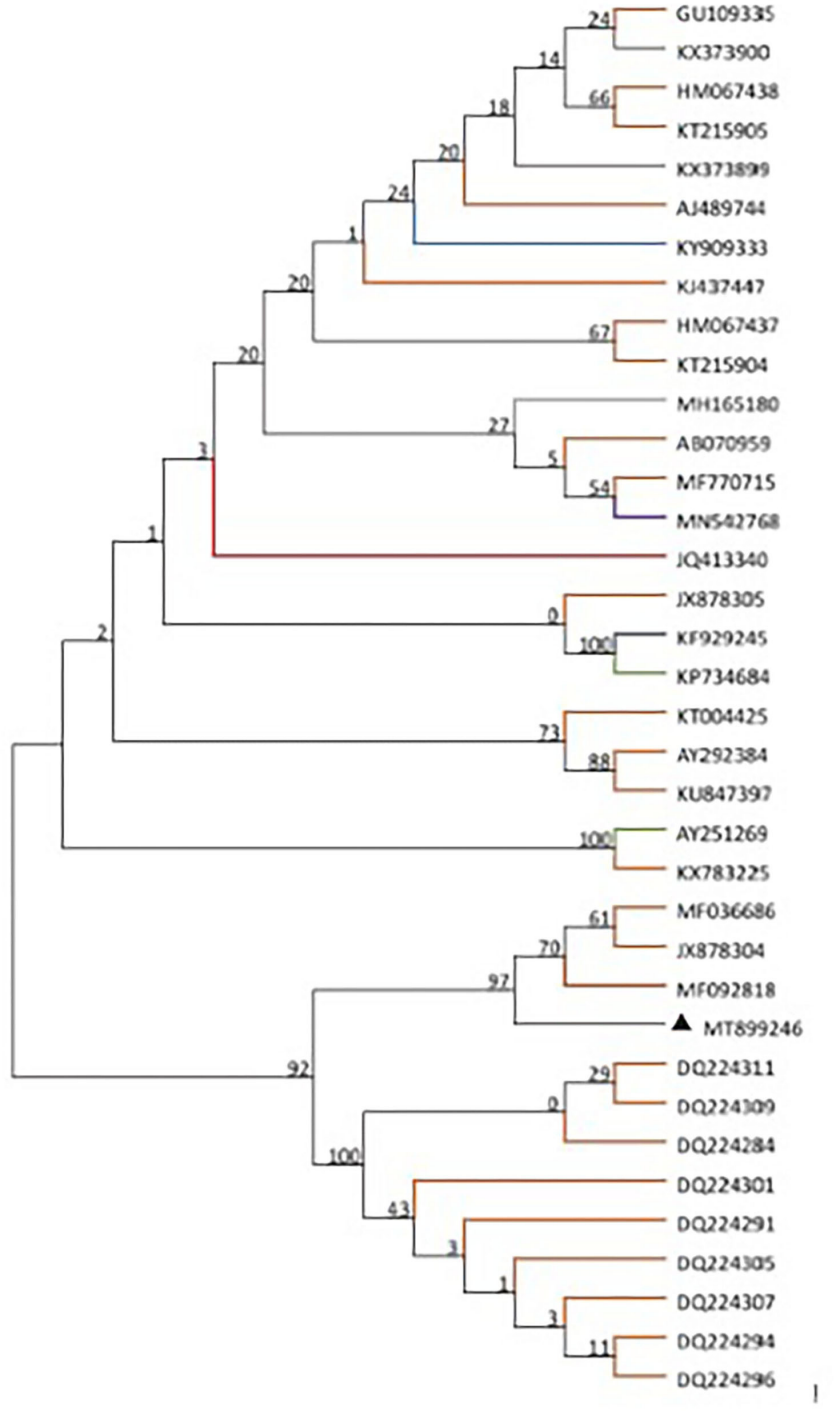
Phylogenetic tree of DWV-A/KV from different insect species including bee, wasp, and ant as well as *Varroa destructor*. The maximum likelihood method (ML) and bootstrap resampling (1,000 repetitions) were used to construct a phylogenetic tree. The number on each branch of the phylogenetic tree represents the bootstrap value (1,000 repetitions), and the branches with different colors indicate different species infected by different DWV strains. The different colors indicate DWV strains from different hosts. *Apis mellifera, Apis cerana, Vespa crabro, Varroa destructor*, vespids wasp, ant, *polistes chinesis, Vespula vulgaris*, stingless bees, bombus, and unknown host are indicated in orange, gray, blue, green, brown, purple, yellow, khaki, red, gray-blue, and black, respectively.

Based on the alignment of amplification fragments of DWV, RdRp, Lp, and VP3 with nucleotide sequences of representative DWV strains from honey bees (*A. mellifera, A. ceranae*), bumblebee, wasp, and a parasite (*Varroa destructor*), phylogenetic analysis was conducted by the neighbor-joining method with 1000 bootstrap replications using MEGA 7.0 (Figure 1). The resulting phylogenetic trees indicated that DWV-A, lp, and RdRp fragments displayed relatively similar phylogenies in different hosts, and DWV strain from stingless bee was close to those of *A. mellifera*, but the VP3 fragment was different from those of lp and RdRp. In the phylogenetic tree of DWV-A/KV, DWV-A/KV of stingless bee was clustered with those of *A. mellifera* and revealed two major groups from China and other countries (Figure 2). These suggested that DWV in stingless bee is close to that of *A. mellifera* no matter whether DWV-A or DWV-A/KV.

## Discussion

The detection results showed that DWV-A or DWV-A/KV occurred in the stingless bee (Apidae sp.), but not DWV-B and C. Previous studies showed that DWV-A and -C were detected in the Brazilian stingless bee *Melipona subnitida* (37), as well as in *Scaptotrigona mexicana* (38), and *Tetragonisca fiebrigi* (39). Our results meanwhile showed that only one genotype, DWV-A or DWV-A/KV, was found in stingless bee Apiade sp, suggesting that the DWV-A genotype was possibly limited in stingless bee Apiade sp from the Yunnan province of China. It can be seen from the evolutionary tree that the bee virus DWV has indeed spilled over to other species.

The current study showed that DWV strains in stingless bee were clustered in the same branch with those of other Asian strains in geographical regions based on the DWV-A, lp, and VP3 nucleotides sequences but not RdRp fragment. Although phylogenetic analysis on DWV-A sequences indicated that DWV-A in Apidae sp. was close to that found in the UK, the other three fragments were close to those of Asia. Similar results were obtained in the phylogeny of DWV-A/KV of Apidae sp. (Figure 2). This suggested that DWV has evolved its specific characterization in genomic sequence during the process of co-evolution with hosts in local regions.

On the other side, although DWV-A was present in stingless bees in China, this DWV-A was not close to that of stingless bees from other regions such as Argentina (39). Our results showed that DWV-A in Apidae sp. was close to that in *A. mellifera* but not to other stingless bee species from other regions. In addition, hornets such as *Vespa crabro* were found to be infected by DWV (40) but our phylogenetic analysis showed that DWV in Apidae sp. was not close to that of *V. crabro*. This indicated that DWV-A of *Apidae* sp. was possibly obtained from *A. mellifera* in China, but further experiments need to be performed.

## Data Availability Statement

The original contributions presented in the study are included in the article/Supplementary Material, further inquiries can be directed to the corresponding author/s.

## Author Contributions

CH conceived this manuscript. LZ, YD, HZ, and MZ performed the experiments. LZ and CH analyzed the data and wrote the manuscript. All authors contributed to the article and approved the submitted version.

## Funding

This research was funded by the Agricultural Science and Technology Innovation Program (CAAS-ASTIP-2021-IAR), the Fundamental Research Funds for CAAS (Y2020PT17), and the Young Talent Program of CAAS, GDAS Special Project of Science and Technology Development (2018GDASCX-0107).

## Conflict of Interest

The authors declare that the research was conducted in the absence of any commercial or financial relationships that could be construed as a potential conflict of interest.

## Publisher's Note

All claims expressed in this article are solely those of the authors and do not necessarily represent those of their affiliated organizations, or those of the publisher, the editors and the reviewers. Any product that may be evaluated in this article, or claim that may be made by its manufacturer, is not guaranteed or endorsed by the publisher.
