# Supplementary material for: Occurrence and Phylogenetic Analysis of DWV in Stingless Bee (Apidae sp.) in China: A Case Report
Source: Front Insect Sci. 2021 Nov 12;1:748074. doi: 10.3389/finsc.2021.748074 (PMC10926549; doi:10.3389/finsc.2021.748074)
Supplement: Supplementary file 1 [file Table_1.docx]

**TABLE S1ǀ** The primers used for DNA viruses detection in this study

| Abbreviation | Primer sequence (5′–3′) | Length |  |
| --- | --- | --- | --- |
| AFB | F：GCTCTGTTGCCAAGGAAGAA | 451bp |  |
|  | R：AGGCGGAATGCTTACTGTGT |  |  |
| EFB | F：GAAGAGGAGTTAAAAGGCGC | 831bp |  |
|  | R：TTATCTCTAAGGCGTTCAAAGG |  |  |
| NSP | F：GGCAGTTATGGGAAGTAACA | 209bp |  |
|  | R：GGTCGTCACATTTCATCTCT |  |  |
| NCER | F：CGGCGACGATGTGATATGAAAATATTAA | 281bp |  |
|  | R：CCCGGTCATTCTCAAACAAAAAACCG |  |  |
| NAPI | F：GGGCATGTCTTTGACGTACTATGTA | 321bp |  |
|  | R：GGGCGTTTAAAATGTGAAACAACTATG |  |  |
| BS1 | F：AAGTCGAACGGGGTGCTT | 976bp |  |
|  | R：TGCACCACCTGTCTCAATGT |  |  |
| CB | F：TGTCTGTGCGGCTAGGTG | 524bp |  |
|  | R：CCACTAGAAGTAAATGATGGTTAGA |  |  |
| AMFV | F：CAGAGAATTCGGTTTTTGTGAGTG | 550bp |  |
|  | R：CATGGTGGCCAAGTCTTGCT |  |  |
| NB | F：TTTATTTTATGTRYACMGCAG | 170bp |  |
|  | R：GACTTAGTAGCCGTCTCTC |  |  |
| AS | F：CGGGAGAATTTGTCCTATCG | 636bp |  |
|  | R：CCCACTTTAACAATCGGGATG |  |  |
| MS | F：TTGCAAAAGCTGTTTTAGATGC | 160bp |  |
|  | R：TGACCAGAAATGTTTGCTGAA |  |  |
| CM | F：CTTTTGACGAACAACTGCCCTATC | 716bp |  |
|  | R：AACCGAACGCACTAAACCCC |  |  |

Note: AFB, American foulbrood; EFB, European foulbrood; NSP, Nosema sp;NCER, Nosema ceranae; NAP,Nosema apis; BS, spiroplasma sp.; CB, crithidia bombus; AMFV, Apis mellifera filamentous virus; NB,Nosema bombi; AS, Apis spiroplasma; MS, mellifera spiroplasma; CM, crithidia mellificae.
