# Supplementary material for: Occurrence and Phylogenetic Analysis of DWV in Stingless Bee (Apidae sp.) in China: A Case Report
Source: Front Insect Sci. 2021 Nov 12;1:748074. doi: 10.3389/finsc.2021.748074 (PMC10926549; doi:10.3389/finsc.2021.748074)
Supplement: Supplementary file 2 [file Table_2.docx]

**TABLE S2ǀ** The primers used for RNA viruses detection in this study

| Abbreviation | Primer sequence (5′–3′) | Length |  |
| --- | --- | --- | --- |
| IAPV | F：AGA CACCAA TCACGG ACC TCAC | 474bp |  |
|  | R：AGA TTTGTCTGTCTCCCA GTGCAC |  |  |
| SBV | F：ATA TACGGTGCGAGA ACTGC | 879bp |  |
|  | R：CTCGGTAATAACGCC ACTGT |  |  |
| DWV | F：GACTGAACCAAATCCGATGTC | 376bp |  |
|  | R：TCTCAAGTTCGGGACGCATTC |  |  |
| DWV-B | F：TGGCTAATCGACGTAAAGCA | 200bp |  |
|  | R：ACTAATCTCTGAGCCAACACGT |  |  |
| CBPV | F：TCAGACACCGAA TCTGAT TAT TG | 560bp |  |
|  | R：ACT ACT AGA AACTCGTCGCTTCG |  |  |
| CSBV | F：CCTGGGAAGTTTGCTAGTATTTACG | 161bp |  |
|  | R：CCTATCACATCCATCTGGGTCAG |  |  |
| ABPV | F：TTA TGTGTCCAGAGACTGTAT | 900bp |  |
|  | R：GCTCCTATTGCTCGGTTT TTC |  |  |
| BQCV | F：TGG TCAGCTCCC ACT ACCTTA AAC | 700bp |  |
|  | R：GCA ACA AGA AGA AACGTA AACCAC |  |  |
| DWV-A/KV | F：GATATGACTGTATCCTCCATAGCATCTC | 396bp |  |
|  | R：GTATGAAACATATGGCACCTCAAAAGTA |  |  |
| LSV | F：TGTAAAACGACGGCCAGTGCCWCGRYTGYTRGTDCCYCC | 577bp |  |
|  | R：CAGGAAACAGCTATGACCGAVGTGGNGGNGCNAGATARAGT |  |  |
| SINV | F：CAATAGGCACCAACGTATATAGTAGAGATTGGA | 253bp |  |
|  | R：GGAATGGGTCATCATATAGAAGAATTG |  |  |
| ALPV | F：GCGTACCATACTACTCACCATATTTATTTA | 140bp |  |
|  | R：AGTTAATCCATAAAGTGCAATCTACAATAC |  |  |

Note: IAPV, Israeli acute paralysis virus; SBV, sacbrood virus; DWV, deformed wing virus; DWV-B, the type B of DWV; CBPV, chronic bee paralysis virus; CSBV, Chinese sacbrood virus; ABPV, acute bee paralysis virus; BQCV, black queen cell virus; DWV-A/KV, the types A of DWV, Kakugo virus; LSV, lake sinai virus; SINV, Solenopsis invicta virus; ALPV, aphid lethal paralysis virus.
