# Supplementary material for: Occurrence and Phylogenetic Analysis of DWV in Stingless Bee (Apidae sp.) in China: A Case Report
Source: Front Insect Sci. 2021 Nov 12;1:748074. doi: 10.3389/finsc.2021.748074 (PMC10926549; doi:10.3389/finsc.2021.748074)
Supplement: Supplementary file 3 [file Table_3.docx]

**TABLE S3ǀ** The primers used in this study

| Abbreviation | Primer sequence (5′–3′) | Length |  |
| --- | --- | --- | --- |
| DWV-A | F：TACTAGTGCTGGTTTTCCTTT | 155bp |  |
|  | R：CTCATTAACTGTGTCGTTGAT |  |  |
| DWV-B | F：TACTAGTGCTGGTTTTCCTTT | 155bp |  |
|  | R：CTCATTAACTGAGTTGTTGTC |  |  |
| DWV-C | F：TACTAGTGCTGGTTTTCCTTT | 152bp |  |
|  | R：CTCATTAACTGAGTTGTTGTC |  |  |
| LP | F：ATTAAAAATGGCCTTTAGTTG | 653bp |  |
|  | R：CTTTTCTAATTCAACTTCACC |  |  |
| RdRp | F：TCCATCAGGTTCTCCAATAACGGA | 450bp |  |
|  | R：CCACCCAAATGCTAACTCTAAGCG |  |  |
| VP3 | F：CCTGCTAATCAACAAGGACCTGG | 355bp |  |
|  | R：CAGAACCAATGTCTAACGCTAACCC |  |  |

Note: DWV-A, the types A of DWV; DWV-B, the types B of DWV; DWV-C, the types C of DWV; LP, L-protein; RdRP, RNA dependent RNA polymerase; VP3, virus protein 3.
