# Supplementary material for: Occurrence and Phylogenetic Analysis of DWV in Stingless Bee (Apidae sp.) in China: A Case Report
Source: Front Insect Sci. 2021 Nov 12;1:748074. doi: 10.3389/finsc.2021.748074 (PMC10926549; doi:10.3389/finsc.2021.748074)
Supplement: Supplementary file 4 [file Table_4.docx]

**TABLE S4ǀ** DWVs strains used in this study

| ID | Genbank | Country | Host | Year |
| --- | --- | --- | --- | --- |
| 1 | AJ489744 | Italy | Apis mellifera | 2002 |
| 2 | AY292384 | USA | Apis mellifera | 2003 |
| 3 | KT004425 | USA | Apis mellifera | – |
| 4 | KX373899 | France | – | 2016 |
| 5 | GU109335 | UK | Apis mellifera | 2009 |
| 6 | KY909333 | Italy | Vespa crabro | 2016 |
| 7 | JQ413340 | Chile | – | 2012 |
| 8 | KU847397 | Austria | Apis mellifera | 2016 |
| 9 | MF036686 | China | Apis mellifera | 2017 |
| 10 | AB070959 | Japan | Apis mellifera | 2001 |
| 11 | JX878305 | Korea | Apis mellifera | 2012 |
| 12 | JX878304 | Korea | Apis mellifera | 2012 |
| 13 | AY251269 | Netherlands | Varroa destructor mites | 2004 |
| 14 | KX783225 | Belgium | Apis mellifera | 2016 |
| 15 | KX373900 | France | – | 2016 |
| 16 | HM067438 | UK | Apis mellifera | 2010 |
| 17 | KT215905 | UK | Apis mellifera | 2015 |
| 18 | KJ437447 | UK | Apis mellifera | 2014 |
| 19 | HM067437 | UK | Apis mellifera | 2010 |
| 20 | KT215904 | UK | Apis mellifera | 2015 |
| 21 | MF770715 | China | Apis mellifera | 2017 |
| 22 | MH165180 | China | Apis cerana | 2018 |
| 23 | DQ224301 | Slovenia | Apis mellifera | – |
| 24 | DQ224291 | Poland | Apis mellifera | – |
| 25 | DQ224305 | Nepal | Apis mellifera | – |
| 26 | DQ224307 | Sri Lanka | Apis mellifera | – |
| 27 | DQ224311 | Canada | Apis mellifera | – |
| 28 | DQ224294 | Germany | Apis mellifera | – |
| 29 | DQ224309 | United Arab Emirates | Apis mellifera | – |
| 30 | DQ224284 | Austria | Apis mellifera | – |
| 31 | DQ224296 | Hungary | Apis mellifera | – |
| 32 | AB242569 | East Asia | Varroa destructor | 2004 |
| 33 | KF929245 | Europe | Bombus lapidarius | 2011 |
| 34 | KP734684 | Europe | Varroa destructor | 2008 |
| 35 | MF092818 | China | Vepids wasp | 2017 |
| 36 | MN542768 | Malaysia | Ant | 2016 |
| 37 | JQ413340 | Argentina | stingless bees | 2015 |
| 38 | MT068464 | New Zealand | Vespula germanica | 2018 |
| 39 | MT068465 | New Zealand | Vespula vulgaris | 2018 |
| 40 | MT068468 | New Zealand | Polistes chinensis | 2018 |
